# Evaluation of Pulmonary Function Tests, Dyspnea Scores, and Antibody Levels at the Six-Month Follow-Up of Patients Hospitalized for COVID-19 Pneumonia

**DOI:** 10.7759/cureus.56003

**Published:** 2024-03-12

**Authors:** Kadir Özmen, Mehmet Meral, Bugra Kerget, Elif Yılmazel Uçar, Leyla Sağlam, Murat Özmen

**Affiliations:** 1 Department of Pulmonology, Erzurum Ci̇ty Hospi̇tal, Erzurum, TUR; 2 Department of Pulmonology, Faculty of Medicine, Ataturk University, Erzurum, TUR; 3 Department of Cardiology, Erzurum City Hospital, Erzurum, TUR

**Keywords:** antibody levels, dyspnea scores, post-covid-19, pulmonary function test, covid-19

## Abstract

Background: Coronavirus disease 2019 (COVID-19) causes various signs and symptoms, especially lung involvement, during acute infection and in the long term. In this study, we evaluated the follow-up results of patients with chronic COVID-19 over a 24-week period.

Methods: The study included a total of 100 post-COVID-19 patients (confirmed by real-time polymerase chain reaction (PCR) of a nasopharyngeal swab) who presented to the post-COVID-19 outpatient clinic with chronic COVID-19 symptoms 12 weeks after diagnosis, between April and June 2021. All of the patients in the study had a history of hospitalization and were grouped based on the severity of the acute COVID-19 infection (moderate: group 1, severe: group 2).

Results: A comparison of pulmonary function test parameters at week 12 showed that forced expiratory volume (FEV1)%, forced vital capacity (FVC)%, diffusing capacity of the lungs for carbon monoxide (DLCO)%, and DLCO divided by the alveolar volume (DLCO/VA)% values were significantly lower in group 2 than in group 1 (p<0.001 for all). At week 24, only DLCO and DLCO/VA values were lower (<0.001 for both). The mean modified Medical Research Council (mMRC) dyspnea scores of groups 1 and 2 were 1.4 ± 0.9 and 2.8 ± 1.1 at 12 weeks and improved to 0.9 ± 0.6 and 1.6 ± 0.6 at 24 weeks, respectively. The groups’ mMRC scores at 12 and 24 weeks differed significantly (p=0.001, p=0.02). There was no difference in levels of IgM and IgG antibodies against severe acute respiratory syndrome coronavirus 2 (SARS-CoV-2) spike protein between the groups at 12 or 24 weeks (p>0.05 for all).

Conclusion: Improvement in pulmonary function parameters and mMRC scores may take longer than 24 weeks, especially in patients with severe COVID-19. Our results indicated that the levels of IgM and IgG neutralizing antibodies did not differ between patients with moderate and severe illness at 12 or 24 weeks.

## Introduction

Coronaviruses are a large family of viruses that can cause mild, self-limiting infections such as the common cold, as well as more serious infections such as Middle East respiratory syndrome (MERS) and severe acute respiratory syndrome (SARS) [1]. Coronavirus disease 2019 (COVID-19) is an acute respiratory disease caused by the novel coronavirus severe acute respiratory syndrome coronavirus 2 (SARS-CoV-2) [2].

As our experience with COVID-19 increases, the effects of acute infection may continue to decrease in the future. The World Health Organization defines the signs and symptoms observed in the first four weeks after COVID-19 infection as acute COVID-19, whereas the persistence of these symptoms for more than 12 weeks after infection is referred to as post-COVID-19 condition [3]. Studies have shown that dyspnea is one of the most common symptoms observed in the post-COVID-19 period, followed by fever, fatigue, myalgia, cough, loss of taste and smell, arthralgia, and post-traumatic stress disorders [4]. One of the most important causes of persistent dyspnea after COVID-19 infection is the affinity of the virus for lung parenchymal tissue [5]. Post-COVID-19 studies have shown that patients with moderate and severe clinical illness have decreased lung capacity and compliance, as well as reduced diffusion capacity [6]. Although the condition resolves spontaneously in most patients, it has led to the administration of treatments such as long-term steroid therapy in some patients, or anti-fibrotic therapies in those who develop parenchymal fibrosis [7]. Especially in patients with severe COVID-19, dyspnea-related functional limitations adversely impact their social lives, and the medications used have also been implicated in the development of comorbidities [8].

SARS-CoV-2 is clinically, genetically, and epidemiologically similar to SARS and MERS, leading to speculation that the detection of anti-SARS-CoV-2 IgM and IgG antibodies may provide insight into the viral infection process [9]. An examination of the antigenic structure of SARS-CoV-2 showed that the body could produce antibodies against the spike (S) and nucleocapsid (N) proteins [10]. It has been observed that IgM antibodies against the S protein peak 10-12 days after disease onset and generally fall below the specified cut-off values within four weeks. In contrast, IgG antibodies were found to peak within two to three weeks and then partially decrease but persist at levels higher than the cut-off values for one to three years [11]. Some studies have shown that antibody levels (especially IgG) in patients with clinically mild COVID-19 infection remain positive for longer, suggesting that these patients may be less susceptible to reinfection [12].

The present study aimed to evaluate and compare changes in symptoms, pulmonary function parameters, radiological findings, and antibody levels in patients recovered from moderate and severe COVID-19 infection over a 24-week follow-up period.

## Materials and methods

**Figure 1:**
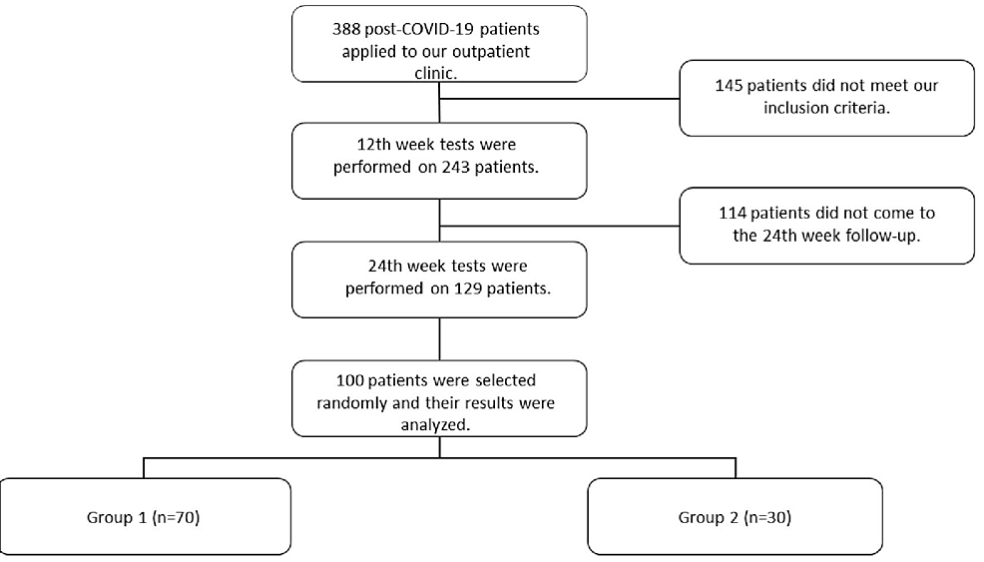
Consort diagram

This prospective study included 100 patients who applied to the post-COVID-19 chest diseases outpatient clinic of the Faculty of Medicine Hospital, Atatürk University, Erzurum, Turkey, between April 2021 and June 2021, had a history of COVID-19 pneumonia, wanted to participate in the study with informed consent, and did not disrupt their follow-up (Figure 1). The study aimed to evaluate the results of clinical, laboratory, and radiological follow-up until 24 weeks after diagnosis. The study was approved by the Atatürk University Faculty of Medicine Ethics Committee. All patients were informed before enrollment about the purpose, methods, and time required for the study. They were also informed that their participation was entirely voluntary, the study involved no risk, and they could withdraw from the study at any time.

Inclusion criteria

The study included patients who had persistent COVID-19 symptoms 12 weeks after being diagnosed as having COVID-19 by real-time polymerase chain reaction (PCR) test of a nasopharyngeal swab. Nasopharyngeal samples of all patients were analyzed by PCR in the Erzurum Public Health Laboratory, Turkey, which has served as the COVID-19 testing center since the start of the pandemic. Inclusion criteria were being over 18 years of age, having radiological findings consistent with COVID-19 pneumonia, not requiring intubation and mechanical ventilation, and agreeing to attend follow-up appointments during the 24-week study period.

Exclusion criteria

Exclusion criteria were the presence of any potential contraindications to pulmonary function testing (recent myocardial infarction, pulmonary embolism, cerebral aneurysm, active hemoptysis, pneumothorax, nausea/vomiting, recent thoracic, abdominal, or ocular surgery) and previously known or newly detected lung pathology unrelated to COVID-19.

Study groups

The patients were divided into two groups based on COVID-19 severity. The patients recovering from moderate illness (nonsevere pneumonia) were included in group 1 (n=70) and the patients who had a history of severe pneumonia (defined as respiratory rate ≥ 30 breaths/min, saturation of peripheral oxygen (SpO2) ≤ 92%, and/or lung infiltration rate > 50%) with intensive care admission due to macrophage activation syndrome or respiratory failure were included in group 2 (n=30).

Study procedure

Patients presenting to the post-COVID outpatient clinic were registered. A detailed history was taken, including smoking history (calculated in pack-years) and the setting of COVID-19 transmission (e.g., family, workplace). Physical examination was performed and patients were asked about symptoms such as fever, dyspnea, cough, fatigue, loss of taste/smell, arthralgia, myalgia, and headache. At follow-up visits, dyspnea scoring was added to symptom questioning. Routine laboratory tests of samples obtained during follow-up visits were evaluated in the biochemistry laboratory of our hospital.

Dyspnea assessment

The modified Medical Research Council (mMRC) dyspnea scale was used for dyspnea scoring [13]. In the mMRC dyspnea scale, the patient selects one of the following statements: I only feel short of breath during high-intensity exercise (0). I get short of breath while walking quickly on level ground or going up a slight slope (1). I walk slower than my peers on level ground because of breathlessness or I have to stop and rest occasionally (2). I get short of breath and have to stop after walking 100 meters or a few minutes on level ground (3). and I am too breathless to leave the house, I get short of breath while dressing and undressing (4). In this study, grade 3 and 4 dyspnea was classified as symptomatic.

Pulmonary function testing

Pulmonary function tests were performed by the technicians in the Erzurum Atatürk University Pulmonary Function laboratory. Spirometry and pulmonary diffusion capacity testing were performed following procedures based on the European Respiratory Society (ERS)/American Thoracic Society (ATS) guidelines [14]. The following parameters were measured: forced vital capacity (FVC), forced expiratory capacity in the first second of exhalation (FEV1), total lung capacity (TLC), and diffusion capacity of the lung for carbon monoxide (DLCO). Hemoglobin level was also measured for DLCO correction. All pulmonary function parameters were expressed as percentages of predicted normal values. Diffusion deficit was defined as a DLCO less than 80% of the predicted value.

COVID-19 Reporting and Data System (CO-RADS) scoring

At initial presentation for acute COVID-19, all patients were assessed radiologically by chest CT and evaluated using the CO-RADS classification [15,16], which is as follows: CO-RADS 1: chest CT is normal, very low likelihood of COVID-19; CO-RADS 2: findings typical of other infections (tree-in-bud sign, centrilobular pattern, lobar or segmental consolidation, cavitation), low likelihood of COVID-19; CO-RADS 3: findings seen both in COVID-19 and other diseases (central ground-glass opacities; interlobular septal thickening or pleural effusion suggestive of pulmonary edema together with extensive homogeneous ground-glass appearance; nodules that are not centrilobular or adjacent to the visceral pleura), moderate likelihood; CO-RADS 4: findings suggestive of COVID-19 (typical findings that are unilateral, are not adjacent to the visceral pleura, show predominantly peribronchovascular distribution, or are superimposed on pulmonary abnormalities), high likelihood of COVID-19; CO-RADS 5: findings typical of COVID-19 (bilateral and multifocal, ground-glass opacities adjacent to the visceral pleura, including fissures, with or without consolidation), very high likelihood of COVID-19; and CO-RADS 6: positive SARS-CoV-2 real-time PCR (RT-PCR), definitive COVID-19 diagnosis [15,16]. Pre-diagnosis CT examinations were evaluated in the Atatürk University Research Hospital Radiology Clinic, and the report made by the radiologist on duty at the hospital that day was taken into consideration when interpreting the results.

Coronavirus Anxiety Scale (CAS)

This scale, developed by Lee in 2020, was used to measure the anxiety levels of the patients in this study [16]. We administered the CAS in our outpatient clinic and determined the Cronbach’s alpha of the scale to be 0.93. The CAS was developed to help identify individuals with impaired functioning due to coronavirus-related anxiety. Diagnostically, the CAS was reported to have 90% sensitivity and 85% specificity. Each item of the CAS is rated on a 5-point scale from 0 (not at all) to 4 (almost every day) based on the respondent’s experiences over the past two weeks. A total score of 9 or higher indicates dysfunctional anxiety associated with COVID-19. A high score on a particular item or high overall score (≥9) indicates that the individual has problematic symptoms that may require further evaluation and/or treatment.

Measurement of serum SARS-CoV-2-specific IgM/IgG antibody

SARS-CoV-2 S protein-specific IgM and IgG antibody levels in serum were measured using COVID-19 IgG detection kits (magnetic beads chemiluminescent immunoassay) purchased from Hunan Yuanjing Biotechnology Co., Ltd, Hunan Sheng, China. Antibody levels were measured in the microbiology laboratory of our hospital. The sensitivity of the kit used to measure antibody levels after 14 days was found to be 99.5% (97.0-100%). For both IgM and IgG levels, values <1 U/mL were accepted as negative and values ≥1 U/mL were considered positive.

Statistical analysis

Based on the results of power analysis using the G*Power program (Heinrich Heine University Düsseldorf, Düsseldorf, Germany), the minimum sample size was determined as 100 for the study to be statistically significant (p=0.05, d=0.50). Data entry and analysis were done using IBM SPSS Statistics for Windows, Version 20, (Released 2011; IBM Corp., Armonk, New York, United States) statistical analysis program. Data were presented as mean, standard deviation, median, minimum, maximum, percentage, and number. Continuous variables were tested for normal distribution using the Shapiro-Wilk W test when the sample size was 50 or larger and the Kolmogorov-Smirnov test when the sample size was smaller than 50. In comparisons between two independent groups, the Student’s t-test was used for normal distributed data, and the Mann-Whitney U test was used for nonnormally distributed data. In comparisons between two dependent groups, the dependent samples t-test and the Wilcoxon test were used for normally and nonnormally distributed data, respectively. Comparisons of continuous variables between more than two dependent groups were done using a general linear model when the data were normally distributed and the Friedman test if nonnormally distributed. Dependent categorical variables were compared using the McNemar test for two groups and the Cochran’s Q test for three or more groups. Correlations were analyzed using Pearson’s or Spearman’s test as appropriate. After analysis of variance (ANOVA), posthoc tests were performed using Tukey’s test when variances were homogeneous and Tamhane’s T2 test when variances were non-homogeneous. Statistical significance was evaluated at an alpha level of 0.05.

## Results

The 100 patients in the study were prospectively enrolled for follow-up between 12 and 24 weeks after COVID-19 diagnosis. No patients were lost to follow-up or were vaccinated against COVID-19 during that time. The patients’ mean age was 55.51 ± 14.75 years and 54% were men. The mean ages of the male and female patients were 58.4 ± 11.42 years and 53.21 ± 10.45 years, respectively. The men in the study were statistically older than the women (p=0.044).

Fifty-seven (57%) of the patients included in the study had at least one comorbidity. Most patients (73%) reported positive family contact, while only 27 patients had a history of positive workplace contact. The mean age of the patients with workplace COVID-19 contact was 42.45 ± 14.78 years, while that of the other patients was 60.23 ± 46 years (p<0.001). The most common symptom at presentation at 12 weeks was fatigue (91%), followed by anxiety (76%) and cough (64%). The patients’ symptoms were significantly improved at 24 weeks (Table 1).

**Table 1:** Evaluation of patients’ symptoms at initial admission for acute COVID-19 and during follow-up for chronic COVID-19. The value at which the p-value is considered significant is p<0.05.

Symptom	At admission		12 weeks		24 weeks		p-value
	Yes (n=100)	No (n=100)	Yes (n=100)	No (n=100)	Yes (n=100)	No (n=100)	
Fever	40	60	0	100	0	100	<0.001
Cough	73	27	64	36	41	59	<0.001
Dyspnea	53	47	53	47	30	70	<0.001
Loss of smell/taste	27	73	4	96	1	99	<0.001
Headache	38	62	50	50	36	64	0.04
Fatigue	57	43	91	9	67	33	<0.001
Arthralgia	35	65	30	70	29	71	0.52
Anxiety	0	100	76	24	58	42	0.001

According to chest CT scans obtained at admission for acute COVID-19, 44% of the patients were CO-RADS category 2, 34% were category 3, 19% were category 4, and 3% were category 5.

Comparison of laboratory parameters and pulmonary function tests between groups 1 and 2 at week 12 (considered the end of the post-acute COVID-19 period) and at week 24 is shown in Table 2.

**Table 2:** Comparison of laboratory parameters and pulmonary function parameters during follow-up between the groups. SD: Standard deviation, FEV1: forced expiratory volume in one second; FVC, forced vital capacity, DLCO/VA, diffusing capacity for carbon monoxide divided by the alveolar volume p*: Between-group comparison of laboratory and pulmonary function parameters at 12 weeks, p**: Between-group comparison of laboratory and pulmonary function parameters at 24 weeks. The value at which the p-value is considered significant is p<0.05.

	12 weeks		24 weeks		p*	p**
	Group 1 (Severe) (n=70) Mean ± SD	Group 2 (Non severe) (n=30) Mean ± SD	Group 1 (Severe) (n=70) Mean ± SD	Group 2 (Non severe) (n=30) Mean ± SD		
WBC (/µL) (N: 3910-10900)	7457.5 ± 1602.1	10179.5 ± 3461.1	7556.7 ± 1428.7	7850.9 ± 1539.7	<0.001	0.38
Lymphocyte (/µL) (N: 1210-3770)	2240.4 ± 693.9	3084.9 ± 1214.1	2521.7 ± 705.7	2596.1 ± 716.9	<0.001	0.64
Fibrinogen (ng/ml) (N: 200-400)	229.9 ± 63.3	338.8 ± 92.8	203.9 ± 47.3	192.3 ± 61.5	<0.001	0.31
Ferritin (ng/ml) (N:22-322)	138.9 ± 141.9	378.1 ± 192.2	112.1 ± 98.6	212.6 ± 111.4	<0.001	<0.001
D-Dimer (ng/ml) (N: 0-500)	471.5 ± 293.8	695.5 ± 295.8	286.8 ± 171.8	419.8 ± 235.1	0.001	0.002
FEV1 (%) (N: ≥80)	112.1 ± 18.7	89.2 ± 18.9	108.8 ± 13.4	106.1 ± 13.3	<0.001	0.46
FVC (%) (N: ≥80)	108.5 ± 20.2	89.4 ± 22.2	100.8 ± 31.2	98.9 ± 22.6	<0.001	0.12
DLCO (%) (N: ≥80)	119.6 ± 22.3	88.7 ± 24.2	114.5 ± 17.7	88.5 ± 16.9	<0.001	<0.001
DLCO/VA (%) (N: ≥80)	116.4 ± 32.4	93.7 ± 20.6	115.7 ± 16.8	93.6 ± 16.1	0.02	0.02

At 12 weeks, WBC, lymphocyte, fibrinogen, ferritin, and D-dimer levels were significantly higher in group 2 than in group 1 (p<0.001, p<0.001, p<0.001, p<0.001, and p=0.001, respectively). A comparison of pulmonary function parameters at 12 weeks showed that FEV1%, FVC%, DLCO%, and DLCO/VA% values were significantly lower in group 2 compared to group 1 (p<0.001 for all). At 24-week follow-up, only D-dimer and ferritin levels were still higher than in group 2 (p=0.002 and p<0.001, respectively), while DLCO and DLCO/VA values were still lower (p<0.001 for both).

The patients’ mMRC dyspnea grades during follow-up are shown in Table 3.

**Table 3:** Comparison of mMRC dyspnea scores of patients at 12- and 24-week follow-ups in the post-COVID outpatient clinic (N=100). mMRC: modified Medical Research Council The value at which the p-value is considered significant is p<0.05.

Description	mMRC dyspnea score	12 weeks n=100 (%)	24 weeks n=100( %)	p-value
Dyspnea during heavy exercise	0	46 (46)	68 (68)	<0.001
Dyspnea when walking quickly on level ground	1	21 (21)	21 (21)	
Dyspnea when walking slower than peers on level ground	2	14 (14)	6 (6)	
Dyspnea after walking 100 meters on level ground	3	17 (17)	5 (5)	
Dyspnea even during daily tasks	4	2 (2)	0 (0)	

The proportion of patients with grade 0 dyspnea (only during strenuous exercise) increased from 46% at three months to 68% at 24 weeks. The distribution of mMRC dyspnea scores changed significantly between 12- and 24-week follow-ups (p<0.001). Post-hoc analysis among groups revealed significant differences between mMRC 0 and 2, between mMRC 0 and 3, and between mMRC 1 and 3 (p=0.002, p<0.001, and p=0.003, respectively). The mean mMRC dyspnea score at 12 weeks was 1.4 ± 0.9 in group 1 and 2.8 ± 1.1 in group 2. At 24 weeks, these scores were 0.9 ± 0.6 and 1.6 ± 0.6, respectively. The groups’ mMRC scores differed significantly at 12 and 24 weeks (p=0.001 and p=0.02, respectively).

Comparisons of the patients’ antibody levels at 12 and 24 weeks are shown in Table 4.

**Table 4:** Comparisons of SARS-CoV-2 IgM and IgG antibodies at 12 and 24 weeks. SD: Standard deviation; SARS-CoV-2: Severe acute respiratory syndrome coronavirus 2 The laboratory's normal range for both IgM and IgG was 0-1 U/ml. p*: Between-group comparison of laboratory and pulmonary function parameters at 12 weeks, p**: Between-group comparison of laboratory and pulmonary function parameters at 24 weeks. Also, the value at which the p-value is considered significant is p<0.05.

	12 weeks		24 weeks		p*	p**
	Group 1 (n=70) Mean ± SD	Group 2 (n=30) Mean ± SD	Group 1 (n=70) Mean ± SD	Group 2 (n=30) Mean ± SD		
IgM (U/mL)	4.8 ± 1.9	5.2 ± 2.7	1.2 ± 0.9	1.4 ± 0.9	0.4	0.3
IgG (U/mL)	6.9 ± 2.2	6.8 ± 2.8	1.8 ± 1.4	1.9 ± 1.5	0.8	0.8

**Figure 2:**
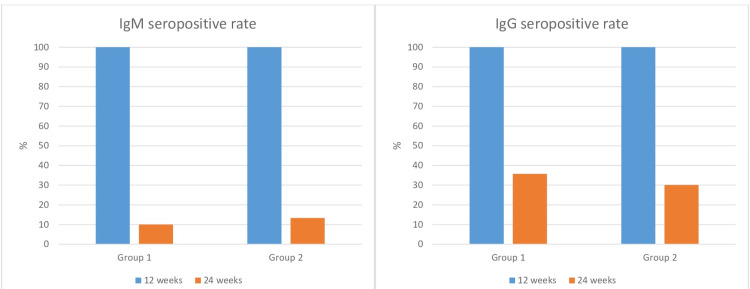
The 12- and 24-week IgM and IgG seropositivity rates of Group 1 and Group 2 patients

In both groups, IgM and IgG levels were significantly lower at 24 weeks compared to 12 weeks. However, there was no statistical difference in IgM or IgG levels between the groups at 12 or 24 weeks (p>0.05). The seropositivity percentages of the groups are shown in Figure 2.

Rates of IgM and IgG seropositivity were 100% in both groups at week 12. At week 24, 10% of patients in group 1 and 13.3% of patients in group 2 were seropositive for IgM, while IgG seropositivity was observed in 35.7% of patients in group 1 and 30% of patients in group 2. There was no difference between the groups in IgM and IgG seropositivity at 12 or 24 weeks (p=0.67 and p=0.7, respectively).

## Discussion

In our study, the main complaint of patients who had a history of inpatient treatment for COVID-19 and presented with chronic COVID-19 was cough, which was replaced by fatigue at 12 and 24 weeks. While the mMRC dyspnea scores of all patients decreased over time, those of patients who had severe acute COVID-19 infection remained higher than those of patients with moderate disease even at 24 weeks. In addition, the pulmonary function parameters of patients with severe acute illness (group 1) had not completely normalized at 24 weeks compared to patients with moderate progression (group 2).

Before COVID-19, post-acute and chronic syndromes were also identified after various inflammatory diseases such as Epstein-Barr virus infection, dengue fever, tickborne encephalitis, influenza, West Nile virus infection, Zika virus infection, and Ross River virus infection. In one study, nearly half of COVID-19 patients evaluated one year after discharge reported persistent fatigue and sleep disturbances (16). Long-term follow-up of people infected with SARS-CoV-2, which is a new strain in the SARS-CoV family, has revealed that some signs and symptoms can persist for a long time. The World Health Organization has defined signs and symptoms lasting for more than 12 weeks after COVID-19 infection as chronic COVID-19 [17]. The most common long-term symptoms of COVID-19 are weakness, shortness of breath, and cough [18,19]. Respiratory symptoms have been attributed to damage to the small airways occurring after the virus infects the lung tissue, and parenchymal fibrosis that results in impaired diffusion capacity.

In addition to using pulmonary function tests to monitor respiratory symptoms in COVID-19 patients, there have also been attempts to assess dyspnea using the mMRC score, which is frequently used in chronic obstructive pulmonary disease (COPD) [20,21]. The results showed that loss of diffusion capacity and high mMRC score in patients with severe acute illness may persist in long-term follow-up. For other observed symptoms, studies have pointed to numerous potential causes of this condition [20,22]. The main factors implicated are sequelae of organ damage, prolonged chronic inflammation, possible autoantibody response, the impact of long-term hospitalization, comorbidities associated with COVID-19 treatment, and possible adverse drug effects. Results from long-term follow-up showed that 26.5% of patients had chronic COVID-19 signs or symptoms [19,23]. In a study sharing the results of a 10-week follow-up after COVID-19, fatigue was reported to be the most common symptom (19).

Researchers have wondered whether the prolonged presence of the SARS-CoV-2 virus in the body and the subsequent constant proinflammatory cytokine discharge may be responsible for the symptoms of chronic COVID-19, and there has also been curiosity regarding the extent to which the body develops immunity against this virus [24]. In particular, there is ongoing debate on the extent of protection offered by IgM and IgG antibodies against S protein, which plays an important role in viral attachment to cells [12]. Different studies have shown that after the onset of symptoms or the first positive PCR result, 9-98% of patients form SARS-CoV-2 S protein-specific IgM, and 15-100% form IgG antibodies [12,25]. While some studies have demonstrated a positive association between disease severity and levels of antibody synthesis, others have indicated that antibody levels do not differ between mild and severe illnesses [26,27].

The rapid increase in the number of COVID-19 cases and the waiting times for PCR tests led to the development of rapid antibody tests [28]. However, in our country, as in the rest of the world, the CO-RADS classification performed after chest CT was used more frequently than rapid antibody tests. In studies on the CO-RADS classification, the false negativity rate was 5.6% (CO-RADS 1, PCR-positive patients), and the false positivity rate was 0.3% (CO-RADS 5, PCR-negative patients). A meta-analysis evaluating the utility of CO-RADS in the diagnosis of COVID-19 based on data from 24 studies showed that CO-RADS scores of 3 and higher had very high sensitivity and specificity in the diagnosis of COVID-19 [29].

The main route of COVID-19 transmission among the patients in our study was domestic contact. In the young adult population, the workplace was another important setting for transmission. Cough was the most common complaint at the initial presentation but was later surpassed by fatigue. Considering the current findings in light of previous studies, this finding may be related to long-term cytokine discharge induced by the virus, as well as to the adverse effects of the medications used during treatment. In the evaluation of laboratory parameters, we observed that many parameters reached the same levels in patients with mild and severe acute COVID-19 within 24 weeks, whereas the positive acute phase reactant ferritin and the fibrin degradation product D-dimer remained higher in patients who had severe illness compared to those whose acute illness was mild. In addition, patients with a history of severe COVID-19 showed lower diffusion capacity in pulmonary function testing. Myofibroblasts are intensively synthesized during the repair of parenchymal damage in patients with severe disease, and despite their important role in the repair of tissue and the interstitial space in the acute period, long-term cytokine discharge can lead to prolonged myofibroblast activity and loss of diffusion in the interstitial space. This may explain the reduced diffusion and elevated acute phase reactant levels observed in our study. In addition, ongoing cytokine discharge may have increased endothelial damage, especially in patients with severe disease, also resulting in an increased D-dimer level. Although dyspnea scores decreased overall in our patients, even at 24 weeks of follow-up, they remained relatively higher in patients who had severe acute illness compared to those with mild illness. The cause of this may be these patients’ limited exertion resulting from reduced diffusion capacity.

In this study, we observed that S protein-specific IgM and IgG levels did not differ statistically significantly between patients with mild and severe acute illness at 12 or 24 weeks. The protective role of anti-S protein antibodies against re-infection remains a subject of debate. While none of the patients followed in our study were re-infected, it is difficult to comment on the protective nature of the antibodies in this regard. Reaching a clear conclusion on this issue would require the same patients to be exposed to SARS-CoV-2 under the same contact conditions and observed, which is not ethically feasible. These antibody levels can only show that antibody positivity occurs at an equal level and duration in all patients, regardless of the course of acute disease.

The retrospective analysis of chest CT scans taken at the time of COVID-19 diagnosis by PCR test suggested that CT had little value in the diagnosis, unlike reported in the literature. We believe that the main reason for this was the unnecessary CT scans that were performed to facilitate quicker patient triage. Considering that radiological signs peak within 7-10 days of symptom onset, we believe that posterior-anterior chest X-ray may be sufficient and more economical for the initial evaluation of the lung parenchyma.

One of the limitations of our study was that CO-RADS staging was evaluated by different radiologists and at different times for each patient. Additionally, other limitations of this study are that it is a single-center study and a quality-of-life questionnaire was not used. As CO-RADS staging is a somewhat subjective evaluation, scores may vary among different radiologists. This is another factor that may further decrease the reliability of CO-RADS staging in the initial period, as seen in our study.

## Conclusions

In conclusion, parenchymal recovery (improvement in radiology imaging) in severe COVID-19 patients may take more than 24 weeks and may manifest itself with low diffusion capacity and a decrease in mMRC score in respiratory function tests. In contrast to literature data indicating high SARS-CoV-2 S spike-specific antibodies in patients with severe illness, we observed no difference in the titers of IgM or IgG antibodies in relation to disease severity in this study. Therefore, both experts and patients should avoid associating disease severity and antibody levels, which may give survivors of severe COVID-19 a false sense of security.
